# In vivo corneal microscopy: from image to insight – where are we going?

**DOI:** 10.1016/j.zemedi.2025.10.001

**Published:** 2025-10-14

**Authors:** Karsten Sperlich, Sebastian Bohn, Florian Worsch, Luisa H. Colorado, Stephan Allgeier, Oliver Stachs

**Affiliations:** a Department of Ophthalmology, Rostock University Medical Center, Rostock, Germany; b Department Life, Light & Matter, University of Rostock, Rostock, Germany; c Department of Optometry, Beuth University of Applied Sciences, Berlin, Germany; d Centre for Vision and Eye Research, School of Clinical Sciences, Optometry and Vision Science, Queensland University of Technology, Brisbane, QLD, Australia; e Institute for Automation and Applied Informatics, Karlsruhe Institute of Technology (KIT), Karlsruhe, Germany

**Keywords:** In vivo confocal microscopy, Corneal imaging, Corneal nerves, Ocular surface, Translational research

## Abstract

In vivo confocal microscopy (IVCM) enables noninvasive imaging of the human cornea at cellular resolution, making it an indispensable diagnostic tool in clinical ophthalmology and biomedical research. The combination of the Heidelberg Retina Tomograph (HRT) and the Rostock Cornea Module (RCM) has established itself as the gold standard for corneal cellular imaging. This review summarizes the imaging principles, technological advancements, and clinical applications of IVCM. It highlights recent advancements, including automated large-area mosaicking of the subbasal nerve plexus, three-dimensional volumetric imaging, time-lapse imaging, and enhanced image analysis techniques. Additionally, corneal immune cell dynamics, critical to understanding corneal pathophysiology, are highlighted. Future challenges and developments aimed at improving diagnostic accuracy and clinical applicability are discussed.

## Introduction

In vivo confocal microscopy (IVCM) is an ophthalmic imaging technique that enables real-time, high-resolution visualization of the living cornea at the cellular or subcellular level. The method provides optical sectioning of corneal tissue, producing histological en face images without requiring tissue removal. Hence, allowing noninvasive, repeatable imaging of the same anatomical structures across multiple time points and enabling longitudinal monitoring of changes associated with disease progression, treatment response, or recovery following a medical or surgical intervention. Since its initial application to the human eye, IVCM has undergone significant technological refinement [1] and has been increasingly adopted in both clinical and research settings. IVCM enables a detailed examination of all corneal layers in both healthy and diseased conditions, offering insights into pathology that complement clinical slit-lamp findings. Subsequent studies further established IVCM as a valuable tool for assessing corneal nerves and other cells across a range of conditions [2]. The growing interest in corneal cellular pathophysiology, together with improvements in confocal imaging technology, has firmly established IVCM as a key diagnostic and investigative modality in modern ophthalmology.

Several types of confocal microscopes have been employed for corneal en face imaging, each with distinct optical principles. The two most widely used systems are scanning-slit confocal microscopes and laser scanning confocal microscopes. The scanning-slit system (exemplified by the Nidek Confoscan series, Nidek Co. Ltd., Japan) uses a vertically oriented slit of light to illuminate the cornea and a conjugate slit for detection, allowing rapid capture of images as the slit scans across the cornea. This design sacrifices some optical sectioning efficiency relative to point-scanning but yields faster image acquisition over a larger area, which is advantageous for screening the cornea with minimal motion artifacts. In contrast, confocal laser scanning microscopy, e.g., the Heidelberg Retina Tomograph combined with the Rostock Cornea Module (HRT-RCM, Heidelberg Engineering GmbH, Germany), utilizes point illumination from a focused laser beam and a pinhole detector, implementing a point-by-point scan of the field of view to build a high-contrast image with excellent axial resolution [3]. Introduced in the early 2000s, the HRT-RCM system provided unprecedented image clarity and has become a workhorse for corneal research and clinical diagnostics [4,5]. The HRT-RCM’s point-scanning approach and use of a water immersion objective yield lateral resolution of around 1–2 μm and axial resolution of ∼4 μm [5,6], enabling the visualization of inflammatory cells and subcellular details, such as epithelial cell borders, cell nuclei of superficial epithelial cells or keratocytes, and dendrites of immune cells.

**Fig. 1:**
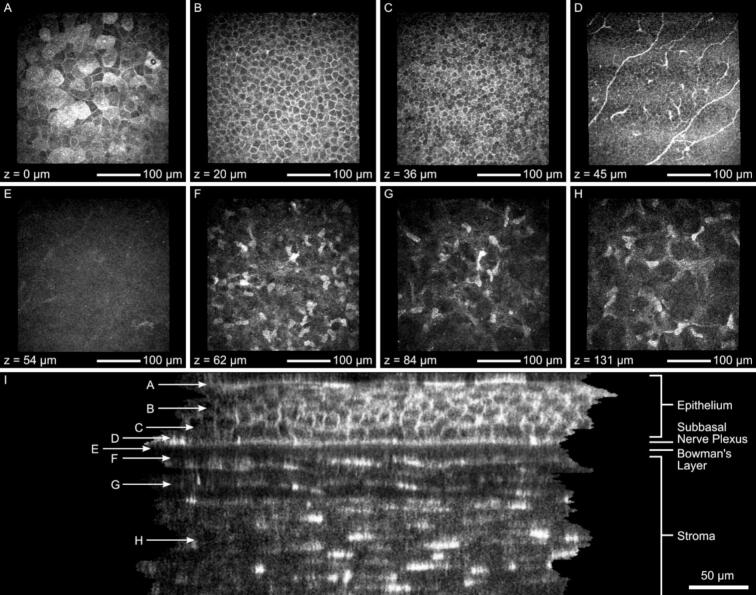
Representative in vivo confocal microscopy scan of the normal human central cornea. (A–H) Sequential en face sections acquired with the in-house developed Rostock Corneal Module 2.0 at the indicated optical depths (z, relative to the epithelial surface). (A) (0 µm), superficial squamous epithelial cells; (B) (20 µm), wing cell layer; (C) (36 µm), basal epithelial cells; (D) (45 µm), subbasal nerve plexus; (E) (54 µm), acellular Bowman’s layer; (F) (62 µm), anterior stroma with highly reflective keratocyte nuclei; (G) (84 µm), mid-stromal keratocytes; (H) (131 µm), posterior stromal keratocytes. (I) Orthogonal reconstruction of the full z-stack (optical section thickness ≈ 1 µm) illustrating the layered cross-section of the cornea. White arrows denote the depths from which images A–H were taken. Adapted with permission from [11] © Optical Society of America.

The principle of confocal imaging offers two major advantages over conventional light microscopy technology: enhanced optical sectioning and improved contrast by rejecting out-of-focus light. As a result, confocal microscopy images of the cornea have much higher contrast and resolution compared to conventional light microscopy of thick tissues [7,8]. Theoretical analyses indicate that confocal microscopy improves both lateral and axial resolution (as defined by full width at half maximum) by a factor of about 1.4 compared to conventional microscopy [9]. Analysis of the optical transfer function shows that the maximum spatial frequency achievable with confocal microscopy is twice that of conventional microscopy [9]. Similarly, experimental measurements of the contrast transfer function show that confocal microscopy achieves up to twice the contrast of conventional microscopes at diffraction-limited resolutions [10]. However, the primary advantage of confocal microscopy is its ability to perform optical sectioning. While in conventional microscopy the background signal of the sample remains constant with increasing defocus, in confocal microscopy the signal decreases and approaches zero [9]. This optical section enables the detailed representation of fine structures in thick tissues. Due to the limited depth of field per image (a few microns), a series of images at successive depths (depth scan or “Z-stack”) can be acquired to obtain volumetric data from which cross-sectional images can be generated. For example, Fig. 1 shows a depth scan acquired with an in-house developed RCM 2.0 [11]. Modern IVCM devices facilitate this by using a motorized focus drive to scan through the cornea, capturing images from the epithelium down to the endothelium [12]. This through-focusing can be performed manually or in automated sequences, and it enables quantitative measurements such as corneal layer thickness. Lateral fields of view for most commercial confocal microscopes are on the order of 400 µm × 400 μm per frame, which, while much smaller than the corneal diameter, is sufficient to sample cellular changes when multiple non-overlapping frames are acquired in a given region [13]. To address the limitations of these smaller frames, techniques for mosaicking or wide-field reconstruction have been developed to create larger composite images of the subbasal nerve plexus and other structures [[14], [15], [16]]. These advances, along with digital image analysis [[17], [18], [19]] and automation [20], continue to enhance the scope and reproducibility of IVCM in both research and clinical practice.

In summary, IVCM of the cornea has matured from a niche experimental technique into a broadly applied imaging modality in ophthalmology. It offers a unique “window” into the living cornea, allowing clinicians to observe pathological changes in situ and researchers to perform longitudinal studies of cellular processes. In the following sections, we review the technological aspects of current IVCM devices, detail the clinical applications of corneal confocal microscopy imaging in various diseases, and highlight emerging experimental and translational uses of IVCM, including its application in systemic diseases and in evaluating novel therapies.

The goal of this review is to provide a comprehensive overview of current technologies and applications of corneal IVCM, including its technical foundations, current device configurations, and recent innovations that potentially enhance its imaging capabilities. Additionally, clinical applications spanning ocular and systemic diseases are highlighted.

## Principles and technology of corneal confocal microscopy

### Optical principles

**Fig. 2:**
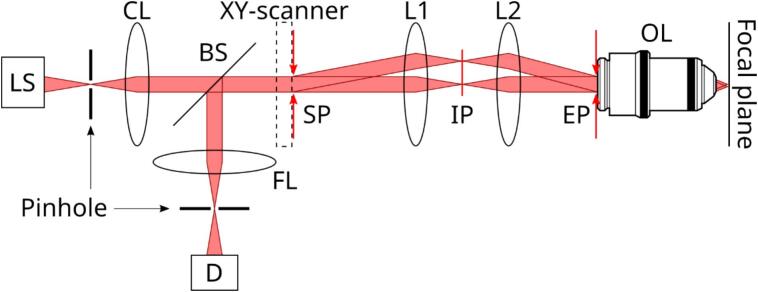
Schematic setup of a confocal laser scanning microscope. A laser source (LS) is spatially filtered by a pinhole and collimated by the collimating lens (CL) before being directed by the beam splitter (BS) toward an XY-scanner (dashed lines). A 4f relay lens configuration (L1 and L2) images the scan pupil (SP) onto both the intermediate image plane (IP) and the entrance pupil (EP) of the objective lens (OL), maintaining conjugate pupil planes during scanning. The OL focuses the beam into the focal plane (right). Reflected light returns through the same optical path, is transmitted by the BS into the detection arm, and is focused by the focusing lens (FL), through a confocal pinhole onto the detector (D). Red rays show the laser light path. Drawing not to scale.

Confocal microscopy uses conjugate focal planes for illumination and detection, confining the imaged volume to the focal point [7,8]. In practical terms, a focused light source (laser or slit) illuminates the tissue, and only light from the focal plane passes through a pinhole to the detector. See Fig. 2 for a schematic representation of a confocal laser scanning microscope. This spatial filtering produces high-contrast, high-resolution images by excluding out-of-focus scattered light. The benefit of corneal imaging is the ability to achieve high-resolution visualization of distinct cellular layers despite the cornea’s transparency and thickness (∼550 μm). IVCM images typically have a narrow depth of field (∼1–5 μm), appearing as thin optical en face sections through the cornea [4]. By incrementally shifting the focal plane (either by moving the objective lens or the sample), a series of images at different depths can be collected, reconstructing the 3D information of the corneal layers [12].

### Confocal microscope configurations

The two main configurations used for corneal IVCM are slit-scanning and point-scanning confocal microscopes. Slit-scanning systems (e.g., Confoscan) use a slit of light to scan rapidly across the cornea, allowing larger area coverage with slightly lower optical sectioning efficiency. Point-scanning systems (e.g., Heidelberg HRT3 with RCM) use a focused point of laser light and a pinhole detector, providing superior axial resolution and contrast at the expense of a slower pixel rate. In a point-scanning system, images are acquired at video rates (∼8–30 frames per second), so small eye movements can cause motion artifacts. To mitigate this, devices use stabilization aids such as special contact caps and software image registration.

### State of the art in IVCM

Because of its superior image quality for most applications, the HRT-RCM has emerged as the state-of-the-art IVCM system. The HRT-RCM is a point-scanning confocal laser microscope optimized for high-resolution in vivo imaging of the human cornea [5]. The HRT was initially conceived as a confocal scanning laser ophthalmoscope for three-dimensional mapping of the optic nerve head. It was extended in 2004 through the integration of the Rostock Cornea Module, transforming the retinal platform into a high-resolution corneal microscope without altering the fundamental optomechanical design [3]. The HRT-RCM is equipped with a 670 nm diode laser as its coherent illumination source. The beam is collimated and passed through a beam splitter into a scanning unit consisting of a resonant galvanometer mirror for fast x-axis (8 kHz scanner resulting in a 16 kHz line rate) deflection and a slower galvanometer for y-axis control. A telescope relay conjugates the scanner pivot point to the back aperture of a high-numerical-aperture water immersion objective (Zeiss Achroplan 63×, NA 0.95), focusing the beam at a working distance of ∼2.2 mm into the cornea.

The system achieves optical sectioning by confining illumination and detection to the same focal volume. Light backscattered from the focal plane is collected along the same optical path, redirected by the beam splitter, and spatially filtered by a confocal pinhole before reaching an avalanche photodiode (APD). This configuration suppresses out-of-focus light and yields lateral and axial resolutions of ∼1–2 μm and ∼4 μm, respectively, which is sufficient to capture cellular structures such as epithelial and endothelial cells, stromal keratocytes, immune cells, and subbasal nerve fibers. Each acquired frame consists of 384 × 384 pixels, covering a nominal 400 μm × 400 μm field of view. Frame rates of approximately 30 Hz enable real-time visualization and live focusing through the full corneal thickness.

The patient interface is designed for stability and reproducibility: the water immersion objective is capped with a sterile disposable polymethylmethacrylate (PMMA) contact element (TomoCap), which is filled with an optically matched coupling gel. After topical anesthesia, the cap is gently applied to the corneal surface, creating a stable optical interface and minimizing axial motion. Patient fixation is guided via an external camera and chin rest assembly, and both confocal and external video feeds are displayed in parallel to ensure proper alignment. Focus adjustment is achieved via a high-precision manual mechanical Z-drive, allowing the operator to scan continuously through the epithelium, stroma, and endothelium. Image acquisition may be performed frame-by-frame or as video sequences, enabling the documentation of static structures and dynamic cellular events.

Overall, the HRT-RCM combines an optoelectronic design with mechanical stability and software control. Its architecture enables reproducible, high-resolution optical sectioning of the living human cornea with histology-like contrast and is extensively used in both clinical diagnostics and translational research applications. Several application examples are mentioned in later sections of this review.

## Current modifications, adjustments, and experimental setups in research

### 3D and wide-field imaging

**Fig. 3:**
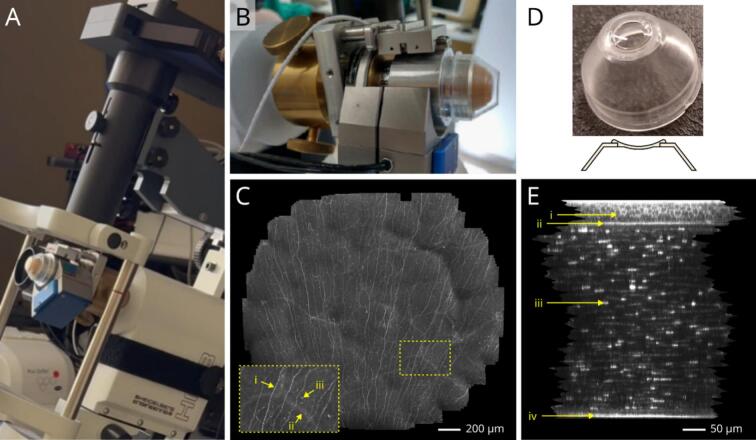
Heidelberg Retina Tomograph (HRT) integrated with EyeGuidance platform and Rostock Corneal Module 2.0 (RCM 2.0). (A) Research setup: Heidelberg Retina Tomograph 3 (HRT 3) base with EyeGuidance stage and the RCM 2.0 scanning head. (B) Close-up of the RCM 2.0 objective fitted with the standard planar polymethyl-methacrylate contact cap. (C) En face EyeGuidance-assisted large-area (4.1 mm^2^) mosaic of the sub-basal nerve plexus, visualizing nerves (i), tissue folds induced by applanation (ii), and immune cells (iii). (D) front view and schematic profile of the custom concave contact cap that matches the corneal curvature and reduces eye movement. (E) Cross-section reconstruction of full corneal thickness obtained from a depth scan (z-stack) acquired with the concave contact cap, visualizing epithelium (i), Bowman’s layer (ii), stromal keratocytes (ii), and endothelium (iv).

Recent advances allow advanced 3D and large-area imaging with confocal microscopy. The in-house developed next-generation “RCM 2.0” and “EyeGuidance” research setup utilizes piezo actuators for a precise and fast focal control and guided eye movement via a moving fixation target to cover a much larger corneal area in continuous image sequences. With an oscillating focal plane and guided eye movements in an outward-growing spiral pattern, this system is capable of continuously scanning a volume of the cornea, computationally classifying tissue layers, and stitching images together in real time, generating a volumetric dataset. An extended en face section mosaic of several mm^2^ can be extracted from such datasets within an image acquisition time of less than a minute. With the aid of a concave contact cap to improve eye stabilization, the system is also capable of reconstructing cross-sectional views of extended depth scans [11]. This wide-field 3D imaging essentially provides in vivo corneal histology: for example, a contiguous mosaic of the subbasal nerve plexus over a few square millimeters can be obtained in minutes, enabling comprehensive assessment of nerve density, tortuosity, and branch patterns that previously required ex vivo analysis [20]. Fig. 3 shows the RCM 2.0 and EyeGuidance system, the concave contact cap, and examples of an SNP mosaic and a cross-section of a full cornea scan.

### OCT-assisted IVCM

A prototype system has combined IVCM with optical coherence tomography (OCT) B-scans to provide simultaneous position and orientation information. This approach utilizes OCT to display the depth and location of the confocal image in real-time, facilitating the targeting of specific layers and the interpretation of confocal findings within their context. OCT-guided confocal imaging has demonstrated improved localization of structures (for example, confirming whether one is imaging Bowman’s layer vs. stroma) and could enhance ease of use in future IVCM devices [21].

Current studies also show that OCT alone is increasingly capable of providing non-contact, in vivo volumetric imaging of the human cornea at cellular resolution [22,23]. While OCT excels in the rapid delivery of cross-sectional views with superior axial resolution, its lateral resolution remains inferior to that of IVCM due to technical limitations. While lateral resolution improves with a higher numerical aperture in both OCT and IVCM, the imaging depth decreases simultaneously in OCT [24]. Thus, OCT inherently involves a trade-off between lateral resolution and imaging depth, which does not apply to IVCM. Additionally, due to the non-contact method and resulting strong surface reflections, the uppermost epithelial layers can only be visualized to a limited extent, if at all.

**Fig. 4:**
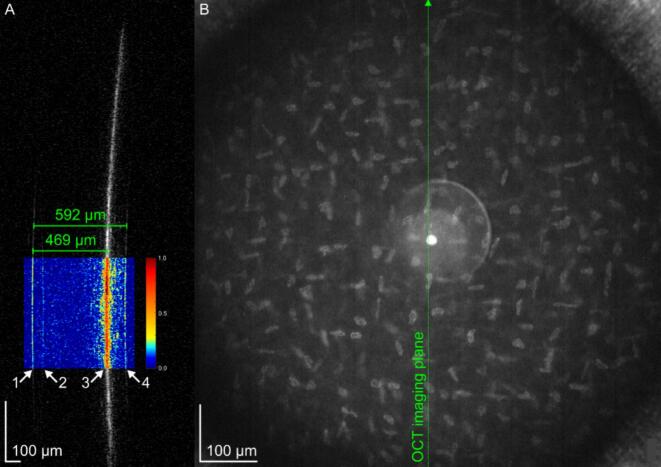
OCT-guided depth targeting during in vivo confocal microscopy. (A) Sagittal anterior-segment OCT-B-scan acquired images simultaneously with IVCM. Four reflective interfaces are seen: (1) Outer surface of the contact cap, (2) OCT system mirror artifact, (3) IVCM imaging focal plane positioned within the posterior stroma, and (4) posterior corneal surface. The green brackets indicated the full central corneal thickness (592 µm) and the axial distance from the anterior surface to the IVCM focal plane (469 µm). Additionally, a selected region is enhanced with a pseudocolor image indicating the signal intensity within the boxed region. (B) The en face IVCM image at the depth marked by the green arrow in (A), showing the posterior stromal keratocyte nuclei. Adapted with permission from [21] © Optical Society of America.

Therefore, OCT-assisted IVCM constitutes a complementary approach by combining the contextual depth and localization of OCT with the cellular-level detail of IVCM. Fig. 4 shows an example of these combined techniques.

### Multiwavelength confocal microscopy

**Fig. 5:**
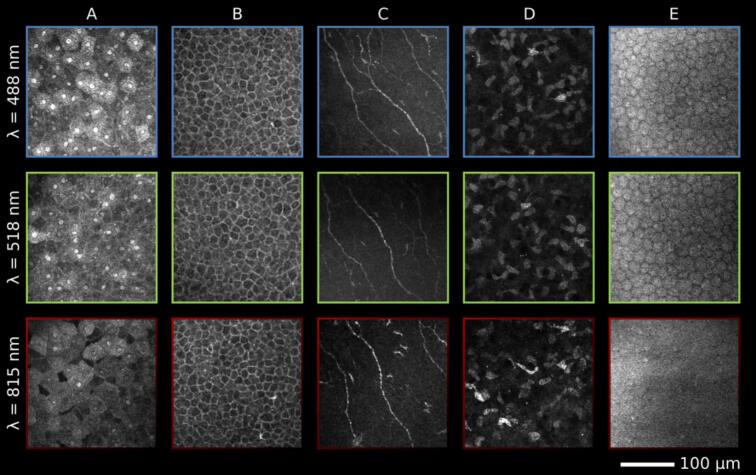
Multiwavelength in vivo confocal images of the normal human cornea. Rows correspond to illumination wavelengths—blue λ = 488 nm (top, blue border), green λ = 518 nm (middle, green border), and near-infrared λ = 815 nm (bottom, red border). Columns show representative layers from anterior to posterior: (A) superficial squamous epithelial cells, (B) basal and wing epithelial cells, (C) subbasal nerve plexus, (D) anterior stroma with keratocyte nuclei, and (E) corneal endothelium. At the three wavelengths, all structures can be recognized similarly well. Only the endothelium (E) can barely be recognized in the near-infrared range. Adapted with permission from [25] © Optical Society of America.

Using different laser wavelengths can slightly alter image contrast and penetration in IVCM. Shorter visible wavelengths (blue-green) provide higher theoretical resolution. They can enhance contrast for superficial cells, whereas longer wavelengths (red/near-IR, like the 670 nm commonly used) penetrate deeper with less scattering. In practice, most clinical IVCM devices use a single red wavelength, which is sufficient for general imaging. However, research systems with selectable wavelengths have shown, for example, crisper epithelial and endothelial detail with blue-green light and cleaner stromal images with red/near-IR [25]. While overall backscatter patterns do not drastically change with wavelength, a tunable multiwavelength confocal microscope could be useful for highlighting certain features (such as inflammatory cells) or optimizing contrast in specific layers. Fig.5 shows an example of different corneal layers at different wavelengths.

### Super-resolution confocal microscopy

Recent advancements in super-resolution confocal microscopy have opened new possibilities for high-resolution IVCM. These techniques enhance conventional point-scanning systems by improving optical resolution and signal-to-noise ratio (SNR) while preserving the optical sectioning required for cellular-level analysis. Foundational principles for these methods were introduced in a work on image scanning microscopy by Müller and Enderlein [26] and have since been adapted to live-cell imaging [27].

One notable development is Airyscan microscopy, which replaces the pinhole detector with a circular array detector [28]. This Airyscan detector utilizes a micro-lens array, where each lens acts as an individual small pinhole, creating individual images with positional information. Each of these images is processed by linear deconvolution followed by pixel reassignment. The final image is created through a weighted summation based on positional information. As a result, the Airyscan achieves approximately a twofold increase in lateral and axial resolution, and an eightfold increase in SNR compared to a conventional pinhole detector, without compromising its optical sectioning capability. This technology is already commercially available in the Airyscan Zeiss 800 microscope series (Carl Zeiss Microscopy Deutschland GmbH, Germany).

Re-scan confocal microscopy represents another super-resolution approach that replaces the single-point detector behind the pinhole with a re-scan unit, which projects the detected light onto a camera, as described by De Luca et al. [29]. This re-scanning is done with a second set of scanning mirrors that is synchronized to the first set at the same frequency and phase. However, the amplitude is adjustable and induces mechanical magnification, which is crucial for improving resolution. A doubling magnification achieves the maximum effect. Here, the distance between two imaged spots doubles, whereas the image of a spot will be blurred by a factor of $\sqrt{2}$, resulting in improved lateral resolution by a factor of $\sqrt{2}$. At the same time, this approach improves the axial resolution by 15 %. Notably, in re-scan confocal microscopy, only the axial resolution depends on the pinhole size, whereas the lateral resolution does not. Now, when compared to a conventional confocal microscope with the same axial resolution, the re-scan confocal microscope detects a stronger signal due to the larger pinhole. Additionally, the stronger focus on sub-resolution objects and the selection of sensitive cameras also contribute to the SNR. De Luca et al. achieved a four times higher SNR with re-scan confocal microscopy and a scientific complementary metal oxide semiconductor (sCMOS) camera compared to a conventional confocal microscope set to the same lateral resolution.

### High-speed confocal imaging technologies

One promising strategy for increasing image acquisition speed in confocal microscopy without compromising spatial resolution is multifocal illumination. A recent study by Suresh et al. implemented a photopolymer-based volume hologram lenslet array illuminator to generate a 9x9 grid of focused illumination spots [30]. This system utilizes a super-Gaussian amplitude profile to ensure consistent illumination across all grid points, preventing intensity fall-off at the periphery. The signal of the multifocal illumination is detected with a charge-coupled device (CCD) camera. The optical magnification is configured so that the distance between adjacent focal spots in the sample and on the CCD camera is sufficient to avoid crosstalk. On the CCD sensor, the focused spots are read out by only a few pixels, so that they act as digital pinholes. The pinhole area and thus the degree of confocality can be adjusted, giving digital control over spatial resolution, optical sectioning, and signal strength. Finally, scanning the illumination grid across the sample, a complete high-resolution image is reconstructed by image stitching. By acquiring data from multiple focal points simultaneously, this method significantly reduces scanning time while maintaining the optical sectioning and spatial resolution of a conventional point-scanning confocal microscope. This makes multifocal approaches highly attractive for dynamic or motion-prone imaging scenarios, such as corneal imaging.

Another novel approach for high-speed, cell-resolving in vivo imaging of the human cornea is chromatic swept-source corneal confocal microscopy (CSS-CCM) [31]. This method combines a swept-source laser with specially designed chromatic optics to replace mechanical axial scanning with fast optical depth scanning. This enables the direct acquisition of cross-sectional images, analogous to slit-lamp views, in real time and at high resolution. CSS-CCM aims to overcome the limitations of current confocal systems by offering reduced motion artifacts combined with subcellular-resolution imaging in clinical and translational ophthalmology.

Please note, the aforementioned super-resolution and high-speed techniques are not yet implemented for in vivo corneal applications.

### Image analysis and quantification

Modern image analysis tools enable quantitative measurements from IVCM images. Software can calculate endothelial cell density, keratocyte density, subbasal nerve fiber length and density, dendritic cell counts, and other metrics [19,[32], [33], [34]]. Subbasal nerve metrics, in particular, are valuable in peripheral neuropathy studies. Researchers often use custom or third-party programs to analyze exported images for more advanced quantification, and machine learning approaches are being developed to identify cells and nerves automatically. A frequently used tool for examining cell morphology is ImageJ, which is freely available to the public [35]. Furthermore, the NeuronJ plug-in is available for ImageJ, which can be used for semi-automatic nerve analysis of SNPs [18,36]. Another frequently used automatic tool for analyzing the SNP is ACCMetrics [17]. These quantitative techniques improve the objectivity and reproducibility of IVCM assessments, supporting its use in both clinical trials and routine practice.

## Clinical applications of corneal IVCM

Corneal confocal microscopy has a broad range of clinical applications due to its ability to detect subtle cellular changes that are invisible to routine examination. It serves as a powerful tool for diagnosing infections, monitoring disease progression, and evaluating treatment effects at the microscopic level.

One of the most important applications of IVCM is in infectious keratitis, where it can often directly identify causative organisms in the cornea. For example, in Acanthamoeba keratitis, a severe parasitic infection often related to contact lens use, IVCM can reveal the parasite’s cysts as bright, double-walled spherical structures (approximately 10–25 µm) in the corneal stroma [37]. Detecting these characteristic cysts with IVCM strongly supports the diagnosis of Acanthamoeba and permits early initiation of anti-amoebic therapy, even before culture results are available. Likewise, in fungal keratitis, confocal imaging may show filamentous fungal hyphae as slender, branching lines in the stroma [38], which is highly suggestive of a filamentous fungal infection and can guide prompt antifungal treatment. IVCM is thus indispensable in ophthalmology for the diagnosis of corneal infections, particularly because it remains the only modality capable of immediately verifying the presence of Acanthamoeba cysts and fungal filaments in vivo and in real-time. This rapid, noninvasive diagnostic capability directly impacts patient outcomes by facilitating the early initiation of appropriate anti-amoebic or antifungal therapy, avoiding the delays associated with culture-based methods, which are often slow and insensitive [39]. A 2023 meta-analysis [40] reported a pooled sensitivity of 94 % and specificity of 87 % for IVCM in detecting Acanthamoeba, underscoring its importance as the only tool that can confirm diagnoses in real-time. For fungal keratitis, the 2023 meta-analysis found that IVCM had a sensitivity of 88 % and specificity of 85 %, indicating its reliability in identifying fungal elements before culture results are available. By contrast, common bacteria are generally too small to visualize directly by IVCM. However, confocal microscopy can still assist in bacterial keratitis by revealing indirect signs such as dense collections of inflammatory cells or certain distinctive patterns (for instance, the branching, rod-like structures that hint at Nocardia). In practice, IVCM provides rapid, in vivo diagnostic clues for challenging corneal ulcers, particularly those likely caused by atypical pathogens, thus enhancing microbiological tests [41].

Beyond infections, IVCM aids in the evaluation of many other corneal conditions. In corneal dystrophies (inherited disorders that cause deposits in various corneal layers), confocal microscopy can visualize the abnormal material in situ and identify which layer is affected. For instance, IVCM can show the lattice-like amyloid strands in lattice corneal dystrophy, the discrete “breadcrumb” granules in granular dystrophy, or the dark, non-reflective holes (guttae) and altered cell mosaic in Fuchs’ endothelial dystrophy [[42], [43], [44]]. This level of detail helps confirm the diagnosis and understand the extent of the disease without the need for an invasive biopsy. In ocular surface inflammatory diseases such as dry eye syndrome, confocal microscopy reveals increased immune cell infiltration (especially dendritic cells in the corneal epithelium and subbasal nerve plexus) and changes in corneal nerves (like reduced fiber density or increased tortuosity), reflecting underlying inflammation and nerve dysfunction [45].

Unlike conventional diagnostic tests, such as Schirmer’s test, tear break-up time, staining, or tear osmolarity, IVCM offers direct visual access to the inflammatory status of the ocular surface. A retrospective cross-sectional study demonstrated that patients with dry eye disease have a significantly increased dendritic cell density (∼93 cells/mm^2^) compared to healthy controls (∼26 cells/mm^2^), along with morphological changes of dendritiform features [46]. IVCM also detects preclinical inflammatory changes that may precede abnormalities on standard tests, offering a window for early diagnosis. This capability is particularly relevant in contact lens wear, where early inflammation may underline lens discomfort or long-term intolerance leading to lens withdrawal. Valencia-Nieto et al. demonstrated that corneal immune cell dynamics measured with time-lapse IVCM correlated with both subjective lens comfort and tear inflammatory mediators, indicating its sensitivity as a potential biomarker for early intervention to preclinical inflammation and patient management strategies [47].

Similarly, in neurotrophic keratopathy, where corneal innervation is impaired due to nerve damage from diabetes, herpes infection, or surgery, IVCM can confirm a decreased nerve fiber density and is used to monitor nerve regeneration during treatments (for example, after administering topical nerve growth factor) [48]. Following corneal surgeries, IVCM is often used to monitor healing at a cellular level. For example, after refractive surgery (LASIK, PRK, SMILE), confocal imaging can document the extent of subbasal nerve fiber loss and its gradual regeneration over months or years [49]. After corneal transplantation, IVCM is helpful to observe the ingrowth of host nerves into the graft and to detect any early immune-mediated changes (like the appearance of dendritic cells signaling potential rejection) [50,51]. In all these scenarios, IVCM provides a unique microscopic perspective that enhances clinical assessment and guides management decisions.

Notably, corneal IVCM has emerged as a useful examination tool beyond the eye, offering insights into systemic diseases. The cornea’s subbasal nerve plexus, composed of small-diameter sensory fibers, is affected by systemic small-fiber neuropathies. As such, IVCM can serve as a noninvasive tool to assess indicators for peripheral neuropathy. A prime example is diabetic peripheral neuropathy: patients with diabetes mellitus often show a significant reduction in corneal nerve fiber density and length on confocal microscopy compared to healthy individuals [52]. This degree of corneal nerve loss correlates with the severity of neuropathy and can even precede clinical symptoms, suggesting a role for IVCM in early detection of diabetic neuropathy [52]. Furthermore, improvements in diabetic control or interventions such as pancreas transplantation have been associated with the regeneration of corneal nerves, as observed on IVCM, indicating that this imaging modality can track nerve recovery over time. For these reasons, corneal nerve parameters measured by IVCM (such as CNFL) are being explored as endpoints in clinical trials for neuropathy treatments [52]. Beyond diabetes, similar corneal nerve alterations have been documented in other neuropathic conditions. For instance, patients with HIV-associated peripheral neuropathy show corneal nerve loss and increased tortuosity [53]. Some patients with neurodegenerative diseases like Parkinson’s disease exhibit reduced corneal nerve density that correlates with disease severity [54]. Even patients undergoing neurotoxic chemotherapy for cancer have been observed to develop corneal nerve and immune cell changes during treatment [55]. Importantly, any insult that disrupts the nervous system can leave a signature in the corneal nerves that is detectable with IVCM. This extends beyond local or autoimmune pathology to include drug–induced neurotoxicity [56], mechanical [57] or surgical trauma [58], and even systemic [59] or psychological [60] conditions have been reported. These findings underscore that the eye can serve as a “window” to systemic health.

Beyond its role in evaluating corneal nerve alterations, IVCM also offers insight into immune-mediated processes and anatomical structures beyond the central cornea. For instance, a study by Bitirgen et al. [61] demonstrated subclinical corneal nerve fiber damage and increased immune cell activation in patients with systemic lupus erythematosus, utilizing IVCM as a noninvasive tool to monitor disease activity. These findings support the idea that the cornea, with its nerve and immune structures, can be used as a surrogate for systemic health.

While IVCM is most often applied to the ocular surface due to the transparency of the cornea and conjunctiva, it can visualize adjacent anatomical structures. For example, the rete ridges of the lid margin share morphological similarities with those in dermal papillae, emphasizing the continuity between ocular adnexal and cutaneous tissues [62]. Notably, the rete ridges are closely associated with Meibomian gland function, underlining their clinical significance.

In summary, IVCM’s clinical applications are diverse, ranging from expediting the diagnosis of corneal infections and dystrophies to quantitatively evaluating neuropathies, providing clinicians with unparalleled insight from real-time, cellular-level imaging of the cornea.

## Experimental and translational applications of corneal IVCM

In addition to its clinical use, IVCM is a valuable research tool in ophthalmology. It allows investigators to study corneal biology and disease mechanisms in living systems repeatedly and at high resolution.

### Use of IVCM in animal models

IVCM permits examination of corneal disease models in animals without sacrificing them at each time point. For example, in a mouse model of corneal nerve injury, serial IVCM tracked the regeneration of subbasal nerves over weeks and showed that treatment with nerve growth factors accelerated nerve regrowth [63]. Similarly, in corneal transplant rejection models, IVCM has visualized host immune cell infiltration and endothelial changes leading up to rejection, providing real-time insight into the graft response [51]. Comparative studies have also imaged the corneas of different species (mouse, rabbit, monkey, etc.), revealing differences in nerve patterns and cell density that help translate animal findings to humans [64].

### Systemic disease models

Since corneal nerve changes mirror those of peripheral neuropathies, IVCM is being utilized in systemic disease research. In diabetic rats, confocal imaging revealed a progressive loss of corneal nerve fibers, corresponding to peripheral nerve damage. Treated diabetic rats (with insulin or neuroprotective drugs) retained more corneal nerves, validating corneal nerves as a biomarker for neuropathy [65]. Likewise, SIV-infected monkeys (an analog of HIV) developed corneal nerve fiber loss similar to that seen in HIV patients, supporting the concept that viral neuropathy affects the cornea [66]. Recently, IVCM has also been applied to monitor corneal changes during systemic chemotherapy in patients. Bohn et al. observed alterations in corneal dendritic cell density over one year of trastuzumab/paclitaxel breast cancer therapy [55], demonstrating that confocal imaging can reflect systemic treatment effects over time. Even in neurodegenerative disease models (such as small-fiber neuropathies in Parkinson’s disease), researchers are examining whether corneal nerve degeneration occurs and if therapies can mitigate it [67]. These studies strengthen the use of corneal IVCM as a bridge between ophthalmology and neurology, confirming that changes observed in patients’ corneas can be reproduced and studied in laboratory models.

### Time-lapse studies

IVCM can be performed repeatedly on the same eye, enabling time-lapse observation of cellular dynamics in vivo. One pioneering human study used continuous IVCM imaging over approximately 50 minutes to track individual immune cells in the cornea of different individuals [68]. It found that some small dendritiform cells migrated rapidly within the cornea at about 1–2 μm per minute [68], confirming active immune surveillance in the normal cornea, while dendritiform cells with long dendrites remained stationary, likely performing surveillance or antigen-presenting roles. Follow-up work showed that dendritiform cell motility slows with age and that it correlates strongly with renal function in diabetes [69]. Sequential IVCM has also been applied to monitor wound healing. For instance, after a corneal abrasion or refractive surgery, daily confocal images documented the influx of inflammatory cells (e.g., white blood cells) to the injury site and their gradual disappearance as the epithelium healed, as well as the apoptosis and subsequent repopulation of stromal cells [70]. By capturing these dynamic changes, time-lapse in vivo confocal studies provide unique insights into processes such as inflammation, tissue repair, and cell migration in the living cornea.

### Therapy evaluation and translational research

Confocal microscopy is invaluable for testing new corneal treatments in both animal models and clinical trials. For pharmacologic therapies, IVCM can detect subtle corneal changes indicative of efficacy or toxicity. For example, in dry eye disease, a successful anti-inflammatory drop might show a decrease in corneal dendritic cell density and improvement in nerve morphology on IVCM. In contrast, chronic use of a preservative-laden medication might be flagged by IVCM findings of reduced subbasal nerve density or epithelial cell changes before patients notice symptoms. For cell-based therapies, IVCM has confirmed that transplanted cells survive and integrate into the cornea [70]. In a clinical trial injecting stem cells into keratoconic corneas, confocal imaging verified an increase in stromal cell density and showed the introduced cells assuming keratocyte-like morphology over months, correlating with improved corneal structure [71]. After limbal stem cell transplantation to treat ocular surface failure, IVCM can noninvasively verify restoration of a healthy corneal epithelium (e.g., noting the presence of normal epithelial cell morphology and the disappearance of invading conjunctival goblet cells) [72]. IVCM is also suitable for detecting early microscopic toxicity in preclinical testing. In rabbits, for instance, it might reveal nerve fiber loss or inflammatory cell infiltration from a candidate drug well before any haze or redness is visible by exam [73,74]. Overall, by providing immediate microscopic feedback, IVCM enables researchers and clinicians to gauge whether a novel therapy is having its intended effect and to alert them to any unintended adverse effects at the cellular level.

## Conclusions and future directions

Corneal IVCM has proven to be a transformative technology in ophthalmic diagnostics and research. This review has outlined how IVCM provides an “inner vision” of the cornea, enabling detection of disease at the cellular level that complements and extends traditional examination methods. Clinically, IVCM enhances our ability to diagnose challenging infections, such as Acanthamoeba and fungal keratitis, at an early stage, to characterize rare corneal dystrophies and deposits in detail, and to monitor corneal health in various conditions, ranging from dry eye disease to the postoperative cornea. The safety and repeatability of IVCM make it particularly useful for following disease progression or response to therapy over time, as evidenced by its use in observing corneal nerve regeneration and immune cell changes longitudinally in patients.

In the research domain, IVCM has established itself as a window into systemic diseases, bridging ophthalmology and neurology by reflecting peripheral neuropathies in corneal nerve metrics. It is now being leveraged as an endpoint in clinical trials (for example, using corneal nerve density as a measure of diabetic neuropathy treatment efficacy) and as a validation tool for novel therapies such as stem cell implants and regenerative drugs. The experimental applications of IVCM are likely to continue to grow as investigators employ confocal imaging in creative ways, such as capturing real-time immune cell movement or assessing the impact of gene therapy on corneal physiology.

Despite the significant progress, challenges remain for the broader implementation of IVCM in routine practice. Interpreting confocal images requires specialized training, and there is a learning curve to accurately identify cell types and subtle abnormalities. Initiatives are underway to improve training, including the development of confocal image atlases and digital teaching sets. Automation via AI may alleviate some of the operator dependency in analysis, providing objective quantifications of nerves and cells that could be utilized by general ophthalmologists with minimal experience. Furthermore, current IVCM devices have a limited field of view and require contact with the eye, which can be uncomfortable for patients and carries a minor risk of corneal abrasion. A major current challenge is the commercial discontinuation of the HRT-RCM, the state-of-the-art IVCM system for corneal imaging in clinical and research settings. As a result, the future of this promising in vivo imaging approach depends on the emergence of clinically applicable next-generation IVCM systems. The chances of this development are that it opens up opportunities to establish new and improved IVCM systems to fill the gap left by the disappearance of the HRT-RCM.

Future technological improvements could include wide-field IVCM systems that image larger corneal areas in a single mosaic for better context, and non-contact confocal systems to eliminate the need for a contact cap, thereby improving patient comfort. There are already experimental approaches combining IVCM with OCT to provide precise localization of confocal images within the cornea, which could enhance its clinical utility.

[31] Another promising direction is integrating IVCM with molecular imaging techniques. For instance, fluorescence confocal microscopy in vivo could enable the specific labeling of cells or pathogens for more definitive identification (currently, clinical IVCM relies on reflectance signals to yield morphological characterization). Researchers are also exploring multi-wavelength confocal microscopy to differentiate cell types by their reflectance spectra, further expanding the information obtainable from in vivo imaging.

In conclusion, in vivo confocal microscopy has established itself as an essential modality for corneal diagnostics and is rapidly evolving into a multidisciplinary tool for medical research. Its contributions span from expediting the diagnosis of blinding corneal infections to offering quantifiable biomarkers for neurologic diseases to guiding cutting-edge regenerative treatments. As technology and analytical methods advance, IVCM will become even more user-friendly and informative. Ongoing developments aim to address current limitations, with wider-field imaging, automated interpretation, and enhanced patient comfort constituting some of the important features to be addressed by next-generation IVCM devices for corneal imaging. With these improvements, IVCM could be more broadly adopted outside of specialty centers, benefiting a larger patient population. Ultimately, the insights gained from examining the living cornea at the microscopic level translate into better patient care by enabling more timely diagnoses, tailored therapies, and improved outcomes in both corneal and systemic diseases. The cornea, through confocal microscopy, will continue to not only provide insights into its own health and disease states but also function as a crucial biometric indicator of the body’s overall health.

## CRediT authorship contribution statement

**Karsten Sperlich:** Writing – review & editing, Writing – original draft, Funding acquisition. **Sebastian Bohn:** Writing – review & editing, Writing – original draft, Visualization, Formal analysis. **Florian Worsch:** Writing – review & editing, Validation. **Luisa H. Colorado:** Writing – review & editing, Visualization, Validation. **Stephan Allgeier:** Writing – review & editing, Validation, Funding acquisition. **Oliver Stachs:** Writing – review & editing, Supervision, Project administration, Conceptualization.

## Declaration of competing interest

The authors declare the following financial interests/personal relationships which may be considered as potential competing interests: Karsten Sperlich: financial support German Research Foundation (DFG grant 469107515)

Karsten Sperlich, Sebastian Bohn and Oliver Stachs: patent applied for chromatic swept-source corneal confocal microscopy (102021214722.4).
